# Retention in Junior Giants, a sport-based youth development program: what factors are associated with intentions to return?

**DOI:** 10.3389/fspor.2024.1360289

**Published:** 2024-04-18

**Authors:** Nicole D. Bolter, Lindsay E. Kipp, P. Brian Greenwood

**Affiliations:** ^1^ Department of Kinesiology, San Francisco State University, San Francisco, CA, United States; ^2^ Department of Health & Human Performance, Texas State University, San Marcos, TX, United States; ^3^ Experience Industry Management Department, Cal Poly San Luis Obispo, San Luis Obispo, CA, United States

**Keywords:** physical activity, developmental sport psychology, youth sport, dropout, parents

## Abstract

**Introduction:**

While evaluation research shows that physical activity-based youth development (PA-PYD) programs can have a positive impact on social and emotional growth, less is known about which participants return year after year and what factors are associated with their continued participation. The Junior Giants is a sport-based youth development program for 5–18-year-old boys and girls that is non-competitive and free to participate. The 8-week program uses baseball and softball as platforms for teaching life skills and fostering social emotional competencies. This mixed-methods study evaluated quantitative factors associated with intentions to return to the program the following year and qualitative reasons why parents/caregivers intended not to re-enroll their child.

**Method:**

Parents/caregivers of Junior Giants participants (*N* = 8,495) completed online surveys about their child's demographics, social emotional climate and learning, character development, and intentions to return the following year.

**Results:**

Descriptive data illustrated that parents/caregivers reported quite positive outcomes and experiences for their child. Chi-square and *t*-test analyses revealed significant differences (*p* < .001) between intended returners (*n* = 7,179, 84.5%) and those who reported no/undecided on returning (*n* = 1,316, 15.5%). Intended returners were significantly more likely to be identified as Latino and be in their second year of participation. Significant predictors of a binomial logistic regression [χ^2^ (*df* = 22) = 1,463.25, *p* < .001] included age, race/ethnicity, years played, character development, reading, league experiences, physical activity, and perceived support, with small to medium effect sizes. Using responses from a subset of 217 parents/caregivers who reported their child would not return to the program, a thematic analysis resulted in seven themes: *Lack of Organization and Communication*; *Dissatisfied with Coaching, Didn't Learn Baseball/Softball, Not Competitive Enough, Skill Levels Not Matched, Aged Out,* and *Non-Program Related Reasons*.

**Discussion:**

Quantitative results contribute to the literature on predictors of retention in youth development programs, while qualitative findings echo common motives cited for dropout in youth sport. Both provide opportunities for reflection and potential changes to future programming.

## Introduction

Physical activity-based positive youth development (PA-PYD) programs are designed to enhance children's and adolescents' social and emotional growth and life skill development by using physical activity and sport as contexts for developing psychosocial and physical skills and behaviors (1, 2). PA-PYD programs can include a variety of structured physical activities (e.g., organized sport, after-school programs) and differ from traditional sport programs because of their intentional goal of nurturing life skills and physical skills simultaneously (2). Much of the evaluation research on PA-PYD has focused on program impact on participants’ psycho-social growth and development (3, 4). However, less attention has been given to understanding participant retention and dropout in these programs. Investigating why participants remain or leave these programs is essential for programmatic changes that will attract and retain as many youth as possible, especially given that these programs have shown to be effective in producing desirable outcomes. The focus of this paper was to examine potential retention and dropout of one sport-based youth development program.

Scholars and practitioners adopting a positive youth development framework focus on how developmental contexts can promote life skill acquisition and personal growth (5–7). This framework positions youth as active agents in their development, rather than problems to be fixed, and has been widely used to understand how programs can be structured for youth to maximize their developmental potential (6). Those who focus on sport and physical activity contexts have seen value in this framework and sought to identify the features needed for positive outcomes to occur, rather than expecting automatic benefits to occur from participation. According to Petitpas et al. (1), sport and physical activity contexts can yield developmental growth provided youth interact with caring, significant adults (external assets) and learn interpersonal and self-management skills (internal assets). Studies have shown a positive impact on youth who participate in PA-PYD programs that have an intentional curriculum and use sport or physical activity as a context to teach life skills and other socio-emotional outcomes (3, 4). For example, programs such as *Teaching Personal and Social Responsibility* (TPSR), *The First Tee,* and *Girls on the Run* have shown favorable outcomes for youth such as life skills transfer, the 5 C's, and increased physical activity levels [e.g., (8–10)]. Overall, results from PA-PYD evaluation studies suggest that engagement in one of these programs can be beneficial to participants, although more rigorous studies are still needed, including longitudinal, intervention, and evaluation designs (11).

To gain expected benefits from these programs, youth must start and continue participating, as Anderson-Butcher (12 p6) suggests, “youth will not benefit if they are not there.” Evidence further suggests that continued participation in these programs can have a sustained impact on participants [e.g., (9, 10, 13)]. In a longitudinal evaluation study, Weiss et al. (9) found that youth in *The First Tee* program showed improvement or stability in life skills transfer over three years of participation. Fewer studies, however, have focused on what factors can predict why youth continue participating. Anderson-Butcher et al. (14) compared returners and non-returners in a sport-based youth development program (LiFE*sports*) geared toward socially vulnerable youth and used pre- and post-camp variables to predict retention status. They found that youth more likely to return had higher fitness levels, greater perceived social responsibility, lower poverty status, and were younger in age, compared to those who did not return. Anderson-Butcher et al. (14) also interviewed parents/caregivers of returners and non-returners, with both groups reporting positive experiences (meeting new people; learning sport skills) and negative experiences (peer conflict) at similar rates. In line with Anderson-Butcher's interpretation and the small effect sizes found, more research is needed to further help predict retention in PA-PYD programs.

Less is also known about why participants might not return to PA-PYD programs (i.e., dropout). Studies examining dropout in youth sport point to several reasons why youth may discontinue participating in their activity. Based on a systematic review of the literature and leisure constraints theory, Crane and Temple (15) determined that youth dropout of sport because of intrapersonal reasons (decreased motivation, change in interest, lack of competence), interpersonal reasons (relationships with peers, teammates, and coaches), and structural reasons (time, money), and that intrapersonal and interpersonal constraints were more often studied. Back et al. (16) conducted a meta-analysis of studies with adolescent team sport athletes and found that aspects related to motivation, along with social support from coaches, parents, and peers, were related to dropout. Of note, these dropout studies have focused on competitive and recreational youth sport environments, so additional research is needed to understand whether these reasons for dropping out are similar or different from sport programs that also have a youth development curriculum.

The focus of this study was on the potential retention and dropout in one sport-based youth development program—the Junior Giants, which is a co-ed baseball and softball program for 5–18-year-olds established in 1994 (17). The program is run through The Giants Community Fund, which is the nonprofit arm of the Major League Baseball team the San Francisco Giants. The 8-week summer program is coached by volunteers, who are primarily parents, and includes one practice and one game per week. The program is non-competitive (i.e., no score kept; no wins/losses) and free. All uniforms and equipment are provided by the Giants Community Fund with the intention that cost is not a barrier to participation. In 2022, over 20,000 youth participated across 81 leagues in underserved communities across California, Nevada, and Oregon.

The Junior Giants curriculum is purposefully designed to promote youth development in the following core elements: health and nutrition including physical activity, anti-bullying, education (focused on offsetting summer reading loss), and character development. A “word of the week” signifies the theme for that week, and coaches and team parents are provided a practice plan that includes baseball/softball drills and strategies for integrating the desired developmental outcomes. The goal is seamless integration of the life lessons in a fun, activity-filled setting, and the Junior Giants curriculum supports those efforts by providing coaches and team parents with specifics on when and how best to incorporate the lessons in the context of the baseball/softball instruction. The full program also includes intentionality by way of social-emotional learning; coaches, team parents, and league leadership are encouraged to promote a variety of self-regulatory competencies, emotional control, and a celebration of diversity, equity, and inclusion. The goal is to promote safety and security for all in a reinforcing and supportive environment. Ideally, doing so will not only result in developmental outcomes for participants in the given year but also will involve a return to participation for subsequent seasons (i.e., retention).

### Purpose

While PA-PYD program impact is certainly important and examined extensively in the research, the purpose of this study was to examine the variables that influence intention to return or dropout. Understanding retention intentions is critical to help youth development organizations maximize the potential for participants to receive sustained developmental benefits and maintain enrollment in these programs. Findings will contribute to a gap in the PA-PYD literature by focusing on retention and dropout and allow for a baseline of comparison for other sport-based youth development programs. Specifically, we compared parent/caregiver perceptions of program characteristics and youth social, emotional, and behavioral outcomes for those who intended to return and those who did not intend to return or were undecided. We also explored open-ended responses about why some parents/caregivers did not intend for their child to return to the program.

## Method

### Participants

The sample (*N* = 8,495) included parents/caregivers of 5–13-year-olds who were currently participating in the Junior Giants program in one of 79 different leagues (see Table 1). Parents/caregivers reported their children were almost 8 years old on average (*M* = 7.93 *SD* = 2.23), and about 70% said their child was in the first year of the program. With baseball being a predominantly male-dominated sport at all levels, Junior Giants offers both baseball and softball as options in a fully co-educational environment wherein all youth regardless of their gender identity can choose to play baseball or softball (for those locations that offer both). Parents/caregivers reported their child's gender identity as follows: Female (33.2%), Male (66.3%), Non-Binary (0.1%), and Preferred Not to Answer (0.4%). Parent reports indicated a diverse sample of youth participants in terms of race and ethnicity: 37.3% Latino, 22.0% Caucasian, 17.7% Multiple Races/Ethnicities, 10.9% Asian, 6.2% Other, 3.8% African American/Black, 1.3% Pacific Islander, and 0.8% Indigenous.

**Table 1:** Descriptives, frequencies, and significance tests for demographic variables.

Variable	Total sample (*N* = 8,495)	Intended returners (*n* = 7,179)	Intended non-returners/ undecided (*n* = 1,316)	*p*-value	Adjusted residual or Cohen’s *d*
	*N* (%) or *M* (SD)	*N* (%) or *M* (SD)	*N* (%) or *M* (SD)		
Age	7.93 (2.23)	7.91 (2.20)	8.07 (2.39)	*p* = .02	*d* = 0.70
Gender^a^				*p* = .72	|.4|
Male	5,631 (66.3%)	4,761 (84.5%)	870 (15.5%)		
Female	2,818 (33.2%)	2,391 (84.8%)	427 (15.2%)		
Non-binary	12 (.1%)	6 (50.0%)	6 (50.0%)		
Prefer not to answer	34 (.4%)	13 (38.2%)	21 (61.8%)		
Race/Ethnicity				*p* < .001	
Latino	3,164 (37.2%)	2,686 (90.6%)	296 (9.4%)		|12.0|
Caucasian	1,869 (22.0%)	1,427 (76.4%)	442 (23.6%)		|11.0|
Multiple races/Ethnicities	1,503 (17.7%)	1,228 (81.7%)	275 (18.3%)		|3.3|
Asian	926 (10.9%)	782 (84.4%)	144 (15.6%)		|.1|
Other	529 (6.2%)	441 (83.4%)	88 (16.6%)		|.8|
African American/Black	325 (3.8%)	276 (84.9%)	49 (15.1%)		|.2|
Pacific Islander	111 (1.3%)	95 (85.6%)	16 (14.4%)		|.3|
Indigenous	68 (.8%)	62 (91.2%)	6 (8.8%)		|1.5|
Years played				*p* = .001	
First year	5,988 (70.5%)	5,005 (83.6%)	983 (16.4%)		|3.6|
Second year	1,390 (16.4%)	1,210 (87.1%)	180 (12.9%)		|2.9|
3 or more years	1,117 (13.1%)	964 (86.3%)	153 (13.7%)		|1.8|

*Note:* Variables in this table represent the child’s demographic characteristics as reported by parents/caregivers.

^a^ All gender categories were reported descriptively but only binary gender categories were used in inferential statistics.

A subset of the sample that reported they did not plan to return to the program the following year (*n* = 217) included responses from 59 leagues. Compared to the overall sample, these children were reported to be slightly older (*M* = 9.04, *SD* = 2.84) and included more Caucasian respondents (35.9%) but a similar gender identity composite (66.4% male).

### Measures

The survey questions aligned with the program curriculum and expected outcomes and were newly developed by the Junior Giants administrators and collaborative researchers for the purpose of program evaluation. These measures also reflected PA-PYD outcomes commonly studied in the literature (18), including confidence and character from Lerner's 5Cs (7), Benson's developmental assets such as integrity and commitment to learning (19), and life skills such as teamwork and leadership (20).

#### Four bases of character development

Four items represented each of the four bases of character development. Parents were asked to “Indicate the level of change you have witnessed in:” followed by one of the four bases of character development (i.e., confidence, integrity, leadership, teamwork). Response options were on a 5-point scale: *significant negative change*, *negative change*, *no change*, *positive change*, *significant positive change.* Items were averaged into one scale and demonstrated internal reliability with the current sample (*α* = .90).

#### Anti-bullying

Three items represented program outcomes related to lessons about anti-bullying attitudes and behaviors. Parents indicated the level of change they noticed in their child (a) being willing to stand up for other kids, (b) showing respect for others, and (c) knowing what to say or do when seeing bullying, with 5 response options ranging from *significant negative change* to *significant positive change.* Items were averaged into one scale and demonstrated internal reliability with the current sample (*α* = .90).

#### League experiences

Three items represented parents'/caregivers' level of satisfaction with aspects of the specific league they participated in. On a 5-point scale ranging from *extremely dissatisfied* to *extremely satisfied*, parents/caregivers reported their perceptions of the (a) league communication, (b) league organization and leadership, and (c) coaches. Items were averaged into one scale and demonstrated internal reliability with the current sample (*α* = .87).

#### Social emotional learning (SEL)

Three items assessed the extent to which their child demonstrated change in their social emotional learning, focusing on self-awareness and self-management competencies more specifically (21). Parents/caregivers reported the level of change witnessed in (a) confidence in their ability to complete tasks, (b) ability to see a task through to completion, and (c) sense of happiness. Parents responded on a 5-point scale ranging from *significant negative change* to *significant positive change.* Items were averaged into one scale and demonstrated excellent internal reliability with the current sample (*α* = .89).

#### Social emotional learning (SEL) climate

Three individual items assessed separate aspects of the perceived social emotional learning climate. Parents/caregivers were asked, “My child feels [accepted; supported; safe and secure] in the program,” with three response options of *never*, *sometimes*, or *always*.

#### Health

Three items focused on any changes parents noticed in their child's behaviors related to physical activity and healthy eating, specifically (a) eating fruits, (b) eating vegetables, and (c) amount of physical activity. Response options included *less*, *same*, or *more*. Each single item was analyzed separately.

#### Education

One item focused on parent/caregiver reports of their children's reading behaviors since participating in the Junior Giants program. Parents/caregivers responded on a 3-point scale (*no*, *same*, *yes*) as to whether their child had “spent more time reading.”

#### Intentions to return

Parents/caregivers were asked, “Will your child play Junior Giants next year?” to assess intentions to return to the program the following season. Response options included *no*, *undecided*, or *yes*. Parents/caregivers who responded *no* were asked an open-ended follow-up question, “why not?” Another open-ended question was asked for general feedback about the program, which helped further explain intentions not to return to the program.

### Procedure

This study was approved by the Institutional Review Board at a public University in California in the United States prior to initiating data collection. All parents/caregivers who had a child participating in the Junior Giants in the summer of 2022 program (*N* = 22,932) were invited to participate in the study at the end of the program. An online survey was created using Alchemer software, and a link was emailed to all parents/caregivers by the Junior Giants central administration. The survey was created in English and translated into Spanish, and each parent/caregiver was initially provided the option of completing an English or Spanish version of the survey. A consent form was also provided on the first page of the survey, and parents agreed to participate in the survey by clicking to the next page. At the end of the survey, parents/caregivers were provided a link to request tickets to a San Francisco Giants game or be entered into a sweepstakes to win San Francisco Giants memorabilia. The survey took about 14 min to complete on average.

### Data analysis

Data were analyzed using SPSS Version 28. Initial screening included missing data analyses and checks for normality. Missing data were considered Missing Completely at Random (MCAR) if Little's MCAR test was non-significant, and values of skewness (<|3|) and kurtosis (<|8|) were considered acceptable (22). Descriptive statistics were run for all continuous variables, while frequencies and percentages were calculated for all categorical variables. Conceptually similar items were combined to create scales and acceptable internal reliability established if Chronbach's *α* > .70. Two groups were used for comparison—intended returners and undecided/intended non-returners. Chi square analyses for categorical variables and *t*-tests for continuous variables were run to identify group differences by intentions to return. Based on the number of planned comparisons (*N* = 15), we used a Bonferroni correction such that statistical significance was established if *p* < 0.003 (0.05/15). For chi square analyses, adjusted residuals >|1.96| were considered statistically significant deviations from expected cell counts (23). Effect sizes for *t*-tests were determined by Cohen's *d* and assessed as small (*d* ≥ .20), medium (*d* ≥ .50), and large (*d* ≥ .80) (24). A binomial logistic regression was run to assess what factors could predict intentions to return (yes vs. no/undecided). Correlations were first run among all predictor variables to check for multicollinearity (*r* > .70). Another Bonferroni correction was used to account for the planned comparisons, where predictors were considered significant at *p* < 0.002 (0.05/24). Odds ratios (OR) were considered small if OR > 1.5, medium if OR > 3.0, and large if OR > 5.0 (25).

The two open-ended questions were analyzed using thematic analysis (26). Phase 1, Familiarization, and Phase 2, Coding, were completed by three researchers who independently read through all responses from intended non-returners and coded relevant data. To begin Phases 3–5 (theme development, refining, and naming), the researchers met to discuss codes and create an initial set of themes. These themes, codes, and raw data were provided for another researcher, who reviewed and provided feedback on the cohesiveness, distinctiveness, and labeling of each theme. The final themes were discussed by all four researchers. To establish credibility, crystallization (27) was achieved by using multiple researcher viewpoints and reaching a complex and in-depth understanding of the data.

## Results

### Quantitative results

Of the 22,932 participants in the program, 37.0% (*N* = 8,495) of parents/caregivers completed the survey. Data screening identified a very small amount of missing data (<.001%) and Little's MCAR test was non-significant (*p* = .251), suggesting data were missing completely at random, so values were replaced using mean imputation. All continuous variables on a 5-point scale suggested normal distributions and acceptable values for skewness and kurtosis, while the seven items with 3-point response options were skewed and kurtotic. Very few parents/caregivers (<.01%) reported their child ate less fruit or veggies or participated in less physical activity; saw no changes in their time spent reading, or perceived their child never felt accepted, supported, or safe and secure. Collapsing response categories is justified in certain situations with compelling evidence, such as a lack of use (28). Given the lack of responses for “never”, “less”, and “no”, responses for “never” and “sometimes” were combined for the three SEL climate items; responses for “less” and “same” were combined for the three health items, and responses for “no” and “same” were combined for the one reading item. Based on these modifications, these items were treated as categorical outcome variables for subsequent analyses.

Descriptive data illustrated that responses from parents/caregivers were quite positive (see Tables 1, 2). On average, parents/caregivers reported significant positive change in their child's character development, anti-bullying strategies, and social emotional learning, and that they were satisfied with their league's organization, communication, and coaches. Most also indicated that their child always felt accepted, supported, and safe and secure. A majority reported their child had increased the amount of physical activity they did and the time they spent reading since participating in the program, while only a third of parents/caregivers said their child ate more fruits or veggies.

**Table 2:** Descriptives, frequencies, and significance tests for program outcomes.

Variable	Total sample (*N* = 8,495)	Intended returners (*n* = 7,179)	Intended non-returners/ undecided (*n* = 1,316)	*p*-value	Adjusted residual or Cohen’s *d*
	*N* (%) or *M* (SD)	*N* (%) or *M* (SD)	*N* (%) or *M* (SD)		
Four bases of character development	4.13 (.54)	4.20 (.51)	3.73 (.55)	*p* < .001	*d* = .91
Anti-bullying	4.02 (.58)	4.09 (.56)	3.68 (.58)	*p* < .001	*d* = .74
Social emotional learning	4.09 (.56)	4.16 (.53)	3.72 (.58)	*p* < .001	*d* = .81
League experiences	4.22 (.78)	4.34 (.66)	3.55 (1.02)	*p* < .001	*d* = 1.08
Time spent reading				*p* < .001	|14.3|
No/same	3,758 (44.2%)	2,939 (78.2%)	819 (21.8%)		
Yes	4,737 (55.8%)	4,240 (89.5%)	497 (10.5%)		
Change in fruit				*p* < .001	|15.3|
Less/same	5,724 (62.1%)	4,192 (79.9%)	1,054 (20.1%)		
More	3,221 (37.9%)	2,970 (92.2%)	251(7.8%)		
Change in veggies				*p* < .001	|12.7|
Less/same	6,135 (72.2%)	4,953 (81.6%)	1,114 (18.4%)		
More	2,360 (27.8%)	2,184 (92.5%)	176 (7.5%)		
Change in PA				*p* < .001	|18.3|
Less/same	2,625 (30.9%)	1,928 (74.2%)	669 (25.8%)		
More	5,870 (69.1%)	5,242 (89.3%)	628 (10.7%)		
Accepted				*p* < .001	|16.9|
Never/sometimes	1,105 (13.0%)	736 (69.0%)	330 (25.1%)		
Always	7,390 (87.0%)	6,435 (87.1%)	955 (12.9%)		
Supported				*p* < .001	|22.5|
Never/sometimes	882 (10.4%)	505 (61.4%)	318 (38.6%)		
Always	7,613 (89.6%)	6,663 (87.5%)	950 (12.5%)		
Safe and secure				*p* < .001	|16.9|
Never/sometimes	539 (6.3%)	315 (61.6%)	196 (38.4%)		
Always	7,956 (93.7%)	6,861 (86.2%)	1,095 (13.8%)		

Tables 1, 2 also show significant differences and effect sizes for intended returners vs. intended non-returners/undecided. Intended returners were significantly more likely to be identified as Latino (*p* < .001) and be in their second year of participation (*p* = .001), while intended non-returners/undecided were more likely to be identified as Caucasian or Multiple Races/Ethnicities and in their first year in the program. There were no significant differences in intentions to return by binary gender identities (male, female) or age. Parents/caregivers who reported their child would return to the program perceived significantly more positive change in all measured program outcomes, with large effect sizes, compared to those who were undecided about returning or definitely not returning.

The binomial logistic regression with all demographic variables and program outcomes as predictors was statistically significant χ^2^ (*df* = 22) = 1,463.25, *p* < .001, and explained almost 28% of the variance (Nagelkerke *R*^2^ = 27.6%) (see Table 3). The model had an overall classification rate of 86.6%, with 98% of intended returners and 23.9% of intended non-returners/undecided classified correctly. Significant predictors included age (*p* < .001), race/ethnicity (*p* < .001), years played (*p* < .001), character development (*p* < .001), reading (*p* = .002), league experiences (*p* < .001), physical activity (*p* < .001), and perceived support (*p* < .001), with small to medium effect sizes. Compared to Caucasian, youth identified as Latino, African American/Black, or Asian were more likely to report intentions to return to the program (2.21 times, 2.21 times, 1.70, respectively). For every unit increase in positive changes in their child's character development (confidence, integrity, leadership, teamwork), parents/caregivers were 2.38 times more likely to report intentions to return to the program. Parents/caregivers were 2.23 times more likely to be intended returners with every unit increase in league satisfaction. Compared to parents/caregivers who reported their child never or sometimes felt supported in the program, parents/caregivers who reported their child always felt supported were 1.56 times more likely to intend on returning.

**Table 3:** Binary logistic regression for intended returners vs. non-intended returners/undecided.

Predictor (reference category)	β	Wald *χ*^2^	*p*-value	Odds ratio	95% CI
Age	−0.11	46.27	<.001	0.90	0.87–0.92
Binary gender (male)	0.05	0.48	0.49	1.05	0.91–1.21
Race/Ethnicity (Caucasian)		84.90	<.001		
Latino	0.79	73.22	<.001	2.21	1.84–2.65
Multiple races/Ethnicities	0.33	11.36	<.001	1.39	1.15–1.68
Asian	0.53	20.72	<.001	1.70	1.35–2.14
Other	0.46	9.50	0.002	1.59	1.18–2.13
African American/Black	0.80	15.39	<.001	2.21	1.51–3.22
Pacific Islander	0.72	5.23	0.022	2.06	1.11–3.82
Indigenous	1.32	6.32	0.012	3.73	1.34–10.40
Years played (first year)		23.40	<.001		
Second year	0.39	15.39	<.001	1.48	1.22–1.79
3 or more years	0.40	12.44	<.001	1.49	1.19–1.86
Four bases of character development	0.87	68.13	<.001	2.38	1.94–2.92
Reading	0.23	9.57	0.002	1.25	1.09–1.45
Anti-bullying	−0.05	0.24	0.62	0.95	0.78–1.16
Social emotional learning	0.15	2.09	0.15	1.16	0.95–1.43
League experiences	0.80	279.77	<.001	2.23	2.03–2.44
Change in fruit (less/same)	0.03	0.08	0.78	1.03	0.84–1.26
Change in veggies (less/same)	−0.24	0.04	0.84	0.98	0.78–1.22
Change in PA (less/same)	0.27	12.56	<.001	1.31	1.12–1.52
Accepted (never/sometimes)	−0.004	0.11	0.97	1.00	0.80–1.24
Supported (never/sometimes)	0.45	11.91	<.001	1.56	1.21–2.02
Safe (never/sometimes)	−0.16	1.19	0.28	0.85	0.64–1.14

### Qualitative findings

Based on thematic analysis, seven themes were created from parents/caregivers' responses: *Lack of Organization and Communication, Dissatisfied with Coaching, Didn't Learn Baseball/Softball, Not Competitive Enough, Skill Levels Not Matched, Aged Out,* and *Non-Program Related Reasons*. These themes are based on responses only from the 217 parents/caregivers who chose “no” to the question about returning the following year and were prompted to elaborate on why not. In the following sections, each theme is discussed, highlighted by relevant participant quotations.

#### Lack of organization and communication

Parents/caregivers noted issues with how the league, practice, and/or games were organized, as well as issues with communication from league administrators and coaches. One participant commented, “It wasn’t as organized as I expected it to be. The kids also never had practice. My son was in another league a year prior and enjoyed it. This year he was very upset.” Another said, “The league was disorganized and unprepared for this season, it hurt the kids the most.” A parent felt disappointed by saying, “Great program but lacking leadership and organizational structure. Emails never get responded to either.”

#### Dissatisfied with coaching

Parents/caregivers were frustrated with either not having a coach or the quality of the coach they did have. Many of these issues seemed to be related to the fact that coaches were volunteers. One parent/caregiver said, “We didn’t have enough coaches for our teams and volunteers were variable,” while another added, “the problem is because it's free and all volunteers, no one takes it seriously.” One parent/caregiver elaborated on how these coaching issues impacted the kids, “Most of these kids had never played before and had no clue what to do or where to go.” This parent/caregiver offered a solution, “Best to have an experienced coach on each team or one that has completed coach training. Since this is volunteer run, time commitment can be a challenge.”

#### Didn't learn baseball/softball

Parents/caregivers felt their child did not learn the skills or rules of baseball or softball. One parent/caregiver simply said, “I don’t feel that my son learned much this year.” Another parent/caregiver said, “It was my daughters first time ever playing and there wasn't that much engagement of teaching how to catch or swing.” Other parents/caregivers worried their children were confused about the rules of the game and perhaps even learning bad habits. One parent/caregiver explained, “Though the league is fun and encouraging, I feel that my player is actually fooling around more and developing some bad habits.” One parent/caregiver suggested, “I would like to see the program reflect more of the actual baseball rules, so that they are learning baseball correctly as well as all the great personal/social growth.” One parent/caregiver explained how skills building should still be important even in a non-competitive environment: “Would have liked to see merit based play a little more. I understand the games are not competitive, but I believe even friendly games can teach excellence and skill building with a structure of competition.”

#### Not competitive enough

Parents/caregivers did not feel the league was at a high enough competitive level for their child. One parent/caregiver noted, “My son plays competitive baseball so it was a step back for him” while another said there was a, “Lack of competitive spirit. Focused more on emotional issues and nutrition instead of the actual game of baseball.” Some parents/caregivers noted that their child wanted to play more competitively, with one writing, “My son is a very competitive person and this isn't the right platform for him,” One parent/caregiver suggested the program provide both competitive and non-competitive opportunities:

Two levels of giants, players who are competitive and want to keep score and play baseball with the rules. And another group who just plays for fun as they do now. I have children who would fit in both categories.

#### Skill levels not matched

Parents/caregivers shared ways in which they felt the skill level of their child was not matched with the other kids on their team or in the league. Some parents/caregivers, for example, reported their child's skill level was superior. One parent/caregiver said, “My son felt this program was remedial to the level of skill he has. I thought the kids would be grouped by their skills.” This parent/caregiver remarked how their child's skill was less than others by saying, “My child felt embarrassed because all her other teammates were much more skilled than her.” Another elaborated on how this mismatch in skill level affected his son:

I think there should be skill levels or divisions based on experience. My son was actually bored at one point because how advanced he was compared to the others. He's had 4 years little league experience and some teammates needed a tee…That's a big difference in skill level.”

#### Aged out

In this theme, parents/caregivers mentioned they would not be returning because their child would be too old to participate the following year. One parent/caregiver said, “He will be 14. If the age is extended, he would return.” Some parents/caregivers said that their child would continue with the program but now as a coach including this one who wrote, “She is going to come back and coach her brothers next year.” One parent/caregiver requested the age range be expanded: “Please expand the age range for older kids who don't necessarily want to play competitive sports but still strive to be a part of a team, feel connected to others their age and stay healthy.”

#### Non-program related reasons

Parents/caregivers cited several reasons they would not be returning to the program that did not directly relate to the program itself. Reasons included traveling during the summer, hot weather, moving out of state, and having “too much going on.” This theme also included parents/caregivers' responses indicating that their child wanted to play a different sport. One parent/caregiver said, “He has decided he wants to focus on a different sport that happens at the same time,” while another commented, “My daughter prefers gymnastics and dance.” Others noted their child was not interested in baseball specifically. One parent/caregiver wrote, “he realized he wasn’t that into baseball” and another noted, “too much downtime for such an active kid.”

## Discussion

This study offers insight into potential retention and dropout of participants in a sport-based youth development program, contributing beyond what is known about PA-PYD program impact that is more often studied. The results were overwhelmingly positive, with most participants saying their child intended to return to the program and very few reporting their child would not come back. Those parents/caregivers reporting intentions to return also perceived a significantly higher positive program impact on their child. This sample represented 37.0% of all participants in the program (8,495 respondents/22,932 total participants), and it is possible that parents/caregivers who had more positive experiences were those who were more likely to complete the survey. Even accounting for potential sampling biases, these results indicate the program is having a positive impact on many participants, which in turn is linked to an increased likelihood of returning to the program. Thus, the program's focus on youth development is not only beneficial for demonstrating program impact but can also lead to better participant retention.

Our results extend what has been previously discovered about retention in PA-PYD programs. Compared to Anderson-Butcher et al.'s (14) study, our results identified more significant predictors with equal or larger effects. Our study focused on intentions to return and used responses from parents/caregivers as predictors, whereas Anderson-Butcher et al. (14) focused on actual retention status (returned vs. did not return) and used data from youth responses. Fredericks and Eccles (29) highlight the importance of parents as providers of children's sport experiences, being the ones who sign them up for programs and decide whether or not they will continue in that activity. The Junior Giants participants represented in our sample were in childhood, when developmentally, parents' beliefs and actions have a significant influence on their sport and physical activity participation (30). Our results support the idea that parents' perspectives may be important for retention for this age group.

The finding that intended return rates were similar for boys and girls is encouraging, even though boys represented two-thirds of all participants. Children's participation in sport declines as they enter adolescence, and dropout is more significant for girls than boys (31). These data suggest the Junior Giants program may be countering this trend and providing a space where girls can and want to continue participating. Furthermore, in the three-year strategic plan for the Junior Giants, one of the priorities is to increase the number of girls in the program from 33% to 50% (32). Demonstrating that parents/caregivers of most girls already participating in the program planned to return is a positive step toward the Junior Giants reaching their goal.

Findings also revealed several factors that differentiated intended returners from those undecided about returning/intended non-returners. For example, youth identified as Latino, African American/Black, or Asian were more likely to report intentions to return to the program compared to youth identified as Caucasian. The explanation for why these differences by race/ethnicity emerged are complex and should be carefully interpreted. Williams and Deutsch (33) outline the problems with using race/ethnicity as a single grouping variable because it ignores the distinctiveness of race, ethnicity, and culture, and does not account for within-group heterogeneity (e.g., racial identity, immigration status, language use). They contend that the lived experiences of race and ethnicity influence how youth interpret the world and shape their beliefs about themselves and others, and the intersection of race/ethnicity with other social categories (e.g., gender, social class) is impossible to disentangle. In our study, it is important to note that we did have relatively lower numbers of participants identified as African American/Black, Pacific Islander, and Indigenous, compared to the other race/ethnicity categories, which may have affected the results.

In addition to demographics, other variables associated with retention were directly related to experiences in the program itself. Parents/caregivers who reported intentions to return also perceived a positive change in their child's demonstration of the Four Bases of Character Development (confidence, integrity, leadership, teamwork). Parents/caregivers expect positive benefits when they enroll their children in a program and correspondingly were happy to report intentions to return when they perceived a return on their investment (29). Perceptions of their child being supported in the program was also a significant correlate and consistent with expected developmental outcomes from PA-PYD programs. Connection, or the relationships that youth develop with their peers and adults, is part of the 5 Cs of youth development (5) and is a key component to the success of PA-PYD programs (5, 10). Finally, whether parents/caregivers were satisfied with the communication, organization, and coaching in the specific league their child participated was also associated with intentions to return. While not commonly cited as a reason for continued participation, these structural issues are certainly cited as reasons for not returning (14). Indeed, our qualitative results pointed to these aspects as important considerations for intended non-returners as well. Parents/caregivers are the logistical drivers of their child's experiences, so if they are satisfied with the communication and organization, it is more likely they would enjoy returning to the program as a parent/caregiver of a participant.

Qualitative responses provided insight into reasons for not wanting to return to the program and aligned with previous research on youth sport dropout (15, 16). Our thematic analysis revealed intrapersonal reasons (not interested in baseball, did not develop skills), interpersonal reasons (issues with coaching, peer comparison in skill level), and structural reasons (too busy in summer to participate, league organization) for why parents/caregivers indicated their child would not return. Most parents/caregivers of non-returners (16 out of 18) in Anderson-Butcher et al.'s (14) study cited a logistical reason for why they did not return (e.g., family schedule conflicts, missed registration), which matches with our theme of *Non-Program Related Reasons*. Thus, our findings suggest that youth discontinue PA-PYD programs and youth sport programs for at least some similar reasons.

The finding that youth may not return to the Junior Giants because they want to participate in other sports is developmentally appropriate. According to the Developmental Model of Sport Participation, youth should try out many different sports in their sampling years (6–12 years old) (34). Evidence suggests that this early diversification of sport experiences can lead to long-term participation in sports and does not impede later success in elite sport. Given the long-term benefits of sport sampling during childhood, the Junior Giants or any PA-PYD program should expect some participants to not return to the program. Moreover, lack of retention because interests have changed, or participants want to pursue other sport activities, should not be viewed as a negative. PA-PYD programs should instead be focused on the reasons for dropout that are within their control (e.g., improving coaching, providing opportunities for skill development).

Two themes—*Didn't Learn Baseball/Softball* and *Not Competitive Enough*—warrant further discussion. Parent/caregiver reports that youth did not develop baseball or softball skills was a little surprising since coaches are provided with detailed practice plans and videos for each week, but also understandable because coaches are volunteers and may not have previous coaching experience. More information about the coaches' experience and adherence to the curriculum is needed to better understand this theme. It is also possible the non-competitive nature of the program may hinder participants' ability to learn the game properly. Torres and Hagger (35) propose that de-emphasizing competition creates confusion for kids and misleads them in understanding what activity they are participating in (e.g., it's called baseball but it's not being played by baseball's rules). This confusion may be heightened for younger children who are more concrete in their thinking (36). On the other hand, the highly competitive nature of youth sport has documented links to stress, burnout, and withdrawal, providing a strong rationale for why de-emphasizing competition is desirable (37). Thinking holistically, PA-PYD programs that have a non-competitive focus need to consider how competition can be de-emphasized while continuing to develop sport-specific skills that prepare youth for future physical activities.

### Limitations

Notwithstanding several strengths of the study (e.g., large sample size, mixed methods), several limitations must be recognized. First, because the sample included 37% of parents/caregivers in the entire Junior Giants network, it is difficult to know whether these perspectives are representative of all program participants. Future evaluation studies should focus on increased recruitment for survey participation to provide a more complete picture of intentions to return to the program or not. Second, all parents/caregivers provided their socioeconomic status during registration but not when they completed the survey at the end of the season. While we know that 68% of players in the entire program receive free or reduced cost lunch at school, we do not know how many of those were represented in this sample. We were thus unable to examine socioeconomic status as a correlate of intentions to return (nor the interactions with race/ethnicity or gender), and future investigations into the Junior Giants would benefit from such analyses. Third, this dataset focused on intentions to return, and future research should include actual retention and dropout data, since intentions do not always translate to behavior (38). It would be additionally interesting to compare intentions vs. actual behavior to identify potential factors or windows as to when and how intentions might change. Fourth, survey questions used in this study reflected content validity (e.g., aligned with program outcomes and previous literature) and showed good internal consistency reliability, and would benefit from additional tests for construct validity (e.g., exploratory factor analysis). Finally, only qualitative responses from non-intended returners were included, and gathering similar open-ended data from parents/caregivers who intended to return and were undecided about returning would be fruitful.

### Practical applications

These results offer practical suggestions for the Junior Giants and their investment in maximizing retention and curbing dropout. For coach training, it can be emphasized that focusing on teaching the four bases of character development and making kids on their team feel supported could be the difference between a kid returning to the program or not. It would also be useful to focus on the transition from the first year to the second year, and perhaps following up more intentionally after their first season to maximize the chances a first-year participant will return to the program. It seems necessary to revisit the curriculum and consider how children of the same age, yet varying skill levels, are accommodated within the same team. Coaches would benefit from resources and training on how to structure practices and learning experiences that support all levels of skill development. An additional emphasis on marketing that the program is non-competitive seems important to avoid mismatched expectations from participants and their parents/caregivers. There may be misconceptions about the program given that the San Francisco Giants, a major league baseball team, is the sponsor of the program itself and parents and kids alike may assume the league is meant to develop elite players.

## Conclusion

Maximizing retention and minimizing dropout are essential to the success of PA-PYD programs. Our findings suggest parents/caregivers may be reliable sources to understand children's intentions to return (or not), and that focusing on youth development goals, such as building character, can increase the likelihood of participants returning. Some reasons for not returning to a program are developmentally expected (e.g., want to try a new sport), while others present opportunities for improvements in programming (e.g., emphasize skill development). While this study focused on the Junior Giants, many findings are transferrable to other PA-PYD programs interested in retaining youth and maximizing their chances of having a sustained impact on participants.

## Data Availability

The dataset presented in this article is not readily available because the data are proprietary to the Giants Community Fund. Requests about the data should be directed to Brian Greenwood, pgreenwo@calpoly.edu.
